# Associations of Mitochondrial Haplogroups B4 and E with Biliary Atresia and Differential Susceptibility to Hydrophobic Bile Acid

**DOI:** 10.1371/journal.pgen.1003696

**Published:** 2013-08-15

**Authors:** Mao-Meng Tiao, Chia-Wei Liou, Li-Tung Huang, Pei-Wen Wang, Tsu-Kung Lin, Jin-Bor Chen, Yao-Min Chou, Ying-Hsien Huang, Hung-Yu Lin, Chao-Long Chen, Jiin-Haur Chuang

**Affiliations:** 1 Department of Pediatrics, Kaohsiung Chang Gung Memorial Hospital and Chang Gung University College of Medicine, Kaohsiung, Taiwan; 2 Department of Neurology, Kaohsiung Chang Gung Memorial Hospital and Chang Gung University College of Medicine, Kaohsiung, Taiwan; 3 Department of Internal Medicine, Kaohsiung Chang Gung Memorial Hospital and Chang Gung University College of Medicine, Kaohsiung, Taiwan; 4 Department of Biological Sciences, National Sun Yat-Sen University, Kaohsiung, Taiwan; 5 Department of Surgery, Kaohsiung Chang Gung Memorial Hospital and Chang Gung University College of Medicine, Kaohsiung, Taiwan; Vanderbilt University, United States of America

## Abstract

Mitochondrial dysfunction has been implicated in the pathogenesis of biliary atresia (BA). This study aimed to determine whether a specific mitochondrial DNA haplogroup is implicated in the pathogenesis and prognosis of BA. We determined 40 mitochondrial single nucleotide polymorphisms in 15 major mitochondrial haplogroups by the use of 24-plex PCR and fluorescent beads combined with sequence-specific oligonucleotide probes in 71 patients with BA and in 200 controls in the Taiwanese population of ethnic Chinese background. The haplogroup B4 and E prevalence were significantly lower and higher respectively, in the patients with BA than in the controls (odds ratios, 0.82 [p = 0.007] and 7.36 [p = 0.032] respectively) in multivariate logistic-regression analysis. The 3-year survival rate with native liver was significantly lower in haplogroup E than the other haplogroups (*P* = 0.037). A cytoplasmic hybrid (cybrid) was obtained from human 143B osteosarcoma cells devoid of mtDNA (ρ^0^ cell) and was fused with specific mtDNA bearing E and B4 haplogroups donated by healthy Taiwanese subjects. Chenodeoxycholic acid treatment resulted in significantly lower free radical production, higher mitochondrial membrane potential, more viable cells, and fewer apoptotic cybrid B4 cells than parental 143B and cybrid E cells. Bile acid treatment resulted in a significantly greater protective mitochondrial reaction with significantly higher mitochondrial DNA copy number and mitofusin 1 and 2 concentrations in cybrid B4 and parental cells than in cybrid E cells. The results of the study suggested that the specific mitochondrial DNA haplogroups B4 and E were not only associated with lower and higher prevalence of BA respectively, in the study population, but also with differential susceptibility to hydrophobic bile acid in the cybrid harboring different haplogroups.

## Author Summary

Mitochondrial dysfunction has been implicated in the pathogenesis of biliary atresia (BA). We determined 40 mitochondrial single nucleotide polymorphisms in different mitochondrial haplogroups in BA patients and controls. The prevalence of haplogroup B4 and E was significantly lower and higher respectively, in the patients with BA than in the controls. The survival rate with native liver was significantly lower in haplogroup E than the other haplogroups. The *in vitro* study using cybrid cells revealed significantly lower free radical production, higher mitochondrial membrane potential, higher mitochondrial DNA copy number and fewer apoptotic in cybrid B4 cells than cybrid E cells. The study provides a novel insight into the etiopathogenesis and the predictive value of mitochondrial haplogroups in BA.

## Introduction

Biliary atresia (BA) is characterized by progressive inflammatory cholangiopathy in infancy. The pathogenesis of BA may involve free radical products derived from mitochondria, resulting in significantly increased superoxide dismutase activity [1]. In the early stage of BA, augmented oxidative DNA and mitochondrial DNA (mtDNA) damage and apoptotic activities were also observed, which was associated with a decreased mitochondrial copy number [2].

The incidence of BA has been reported to be high in some geographic areas, including French Polynesia and Taiwan [3]–[5]. An interesting finding from a mitochondrial lineage analysis revealed that a specific mitochondrial haplogroup was associated with particular aboriginal races in Polynesia and Taiwan [6], with a rapid dispersal of maternal lineages from Taiwan approximately 4000 years ago [7]–[9]. Mitochondria are maternally inherited and accumulate mutations faster than does nuclear DNA [6]. An analysis of normal human mitochondrial DNA (mtDNA) identified many haplogroup-specific patterns of polymorphisms that have arisen over the last 150,000–200,000 years [10]–[12]. Studies in the past 10 years have identified an association of mtDNA single-nucleotide polymorphisms with many diseases [13]–[15]. The mtDNA mutation and mtDNA haplogroups are important in understanding the etiology and pathogenesis of human disease [16]–[18], as mutations of the mitochondrial D-Loop sequence have been demonstrated to be a risk factor for hepatocellular carcinoma (HCC) development [18].

In the present study, we identified the importance of the association of haplogroups B4 and E with BA, which confer resistance or susceptibility to hydrophobic bile acid and possibly to the different incidence of BA in different populations.

## Results

### Association of BA with specific mitochondrial haplogroups

The demographic data of 71 patients with BA, 52 children and 148 adults as controls, are shown in Table 1. Except for age, both control groups were devoid of liver diseases and their genetic distributions were almost identical except for one case of haplogroup E in the adult controls. It was therefore put together in the following statistical comparison of haplogroup prevalence with the patients with BA.

**Table 1:** Descriptive data of studied population.

Variable	BA	Control (children)	Control (Adult)
number	71	52	148
age median (range years)	2.7 (0.1–11)	2.0 (0.3–16)	41 (18–60)
mean ± SD (years)	3.6±4.6	4.4±5.0	40.3±8.8
sex (male/female)	35/36	25/27	65/83
liver disease	71	0	0
haplogroup B4	0	5	18
haplogroup E	4	0	1
liver transplantation	49	0	0
liver transplantation mean ± SD (years)	3.9±4.7	0	0
total deaths (with transplant)	7 (3)	0	0

BA: biliary atresia.

There were 4 cases of haplogroup E, all received kasai portoenterostomy. However, none survived with native liver. Among them, 3 received liver transplantation at ages 8 months, 1 year 5 months, 1 year 10 months, and the other one died at 3 years 1 month of age without receiving liver transplantation. The 3-year survival rate with native liver was significantly lower in haplogroup E than the other haplogroups (25.0% *vs* 48.6%, *P* = 0.037).

Evaluation of haplogroup distributions revealed that the haplogroup B4 was less prevalent in patients with BA than in the control group (odds ratio [OR], 0.71; 95% confidence interval [CI], 0.66–0.77; p = 0.001). In contrast, haplogroup E was more prevalent in BA than in the control group (OR, 11.88; 95% CI, 1.31–108.16; p = 0.018; Table 2). Multivariate logistic-regression analysis of haplogroups associated with BA with adjustment for age and sex, showing the risk for haplogroup B4 was p = 0.007, OR 0.82, 95%CI 0.71–0.85 and for haplogroup E was p = 0.032, OR 7.36, 95%CI 1.22–94.6.

**Table 2:** Association between biliary atresia (BA) and the mitochondrial haplogroups.

	BA		Control				
Haplogroups	Positive	Negative	Positive	Negative	OR	95% CI	p
M10	2	69	4	196	1.42	0.25–7.93	0.654
M7	11	60	32	168	0.96	0.45–2.03	1.000
M8	5	66	10	190	1.44	0.48–4.37	0.549
E	4	67	1	199	11.88	1.31–108.16	0.018*
G	1	70	10	190	0.27	0.03–2.16	0.298
M9	4	67	4	196	2.93	0.71–12.02	0.213
M8&Z	0	71	5	195	1.36	1.27–1.47	0.331
Z	1	70	1	199	2.84	0.18–46.06	0.456
C	1	70	1	199	2.84	0.18–46.07	1.456
B4	0	71	23	177	0.71	0.66–0.77	0.001*
B5	2	69	14	186	0.39	0.09–1.74	0.253
F	13	58	48	152	0.71	0.36–1.41	0.324
A	4	67	13	187	0.86	0.27–2.73	1.000
Y	0	71	1	199	1.36	1.26–1.46	1.000
D	2	69	0	200	3.90	3.18–4.78	0.068
D4	6	65	24	176	0.68	0.27–1.73	0.512
D5	4	67	18	182	0.60	0.20–1.85	0.372
N9	2	69	7	193	0.80	0.16–3.94	1.000

BA: biliary atresia, *p<0.05.

### The main DNA sequence difference(s) between mtDNA of B4 and E

The main differences between our mtDNA B4 and E are: T3027C (16SrRNA), G4491A (ND2, V 8 I), G7266T (COI, S 455 A), G7598A (COII, A 5 T), A7934G (COII, I 117 V), A8701G (ATPase6, T 59 A), A10398G (ND3, T 114 A), C10400T (ND3, T 114 A), T14577C (ND6, I 33 V), T16217C (D-loop), detected following a full-length sequencing of haplogroups B4 and E, shown in Table S1.

### Differential mitochondrial membrane potential response to bile acids in cybrid B4 and E cells

Before conducting the experiments on different cybrids, we confirmed the absence of Cox-II in 143B ρ^0^ cells depleted of mtDNA but not in the wild-type 143B and cybrid B4 and E cells (Figure S1A). Organic anion-transporting polypeptides (OATPs) are sodium-independent organic anion transporters found in a variety of tissues, including those of the liver, and in the studied 143B cells. OATPs contribute to the transport of bile acids. To assess the responsiveness of the cybrids to bile acid treatment, we identified the bile acid receptor OATP mRNA (Figure S1B) and protein expression in the cybrids (Figures S1C and D).

**Figure 1:**
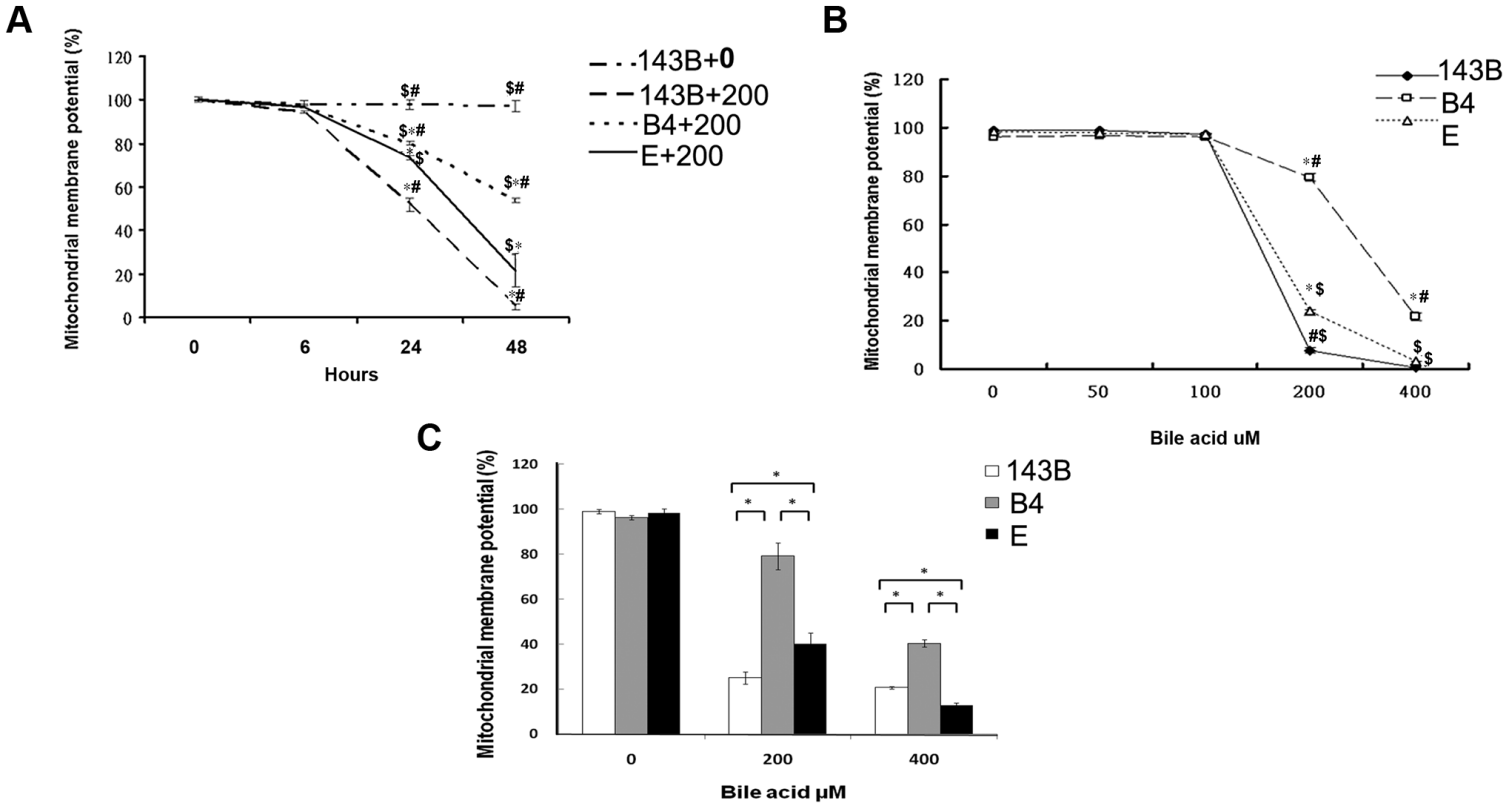
Time- and dose-dependent mitochondrial membrane potential in the parental 143B cells, and cybrid B4 and E cells in response to the bile acid treatment. (A) Time-dependency of mitochondrial membrane potential in parental 143B cells, B4 and E cybrids in response to 200 uM of bile acid. * P<0.001 compared to parental 143B cell+0 uM of bile acid at the same times. **#** P<0.001 compared to cybrid E+200 uM of bile acid, $ P<0.001 compared to cybrid 143B+200 uM of bile acid at the same times. (B) dose-dependency of mitochondrial membrane potential in parental 143B cells, B4 and E cybrids in response to different concentrations of bile acid, ranging from 0 uM to 400 uM. * P<0.001 compared to parental 143B cell at the same doses of bile acid. # P<0.001 compared to cybrid E at the same doses of bile acid. $ P<0.001 compared to cybrid B4 at the same doses of bile acid. (C) Mitochondrial membrane potential in parental 143B cells, B4 and E cybrids. The results represented were mean ± SE in six times tests. * P<0.05 compared at the same doses of bile acid.

In comparison with the wild-type 143B cells without bile acid treatment, the 143B cells and cybrid B4 and E cells treated with bile acid had a significantly decreased mitochondrial membrane potential (MMP). The change was time- and dose-dependent in all the 3 cell lines (Figures 1A and B). MMP was significantly higher in the cybrid B4 cells than in the cybrid E and parental 143B cells (Figure 1C).

### Difference in reactive oxygen species production and mitochondrial copy number between the cybrid B4 and E cells in response to bile acid treatment

**Figure 2:**
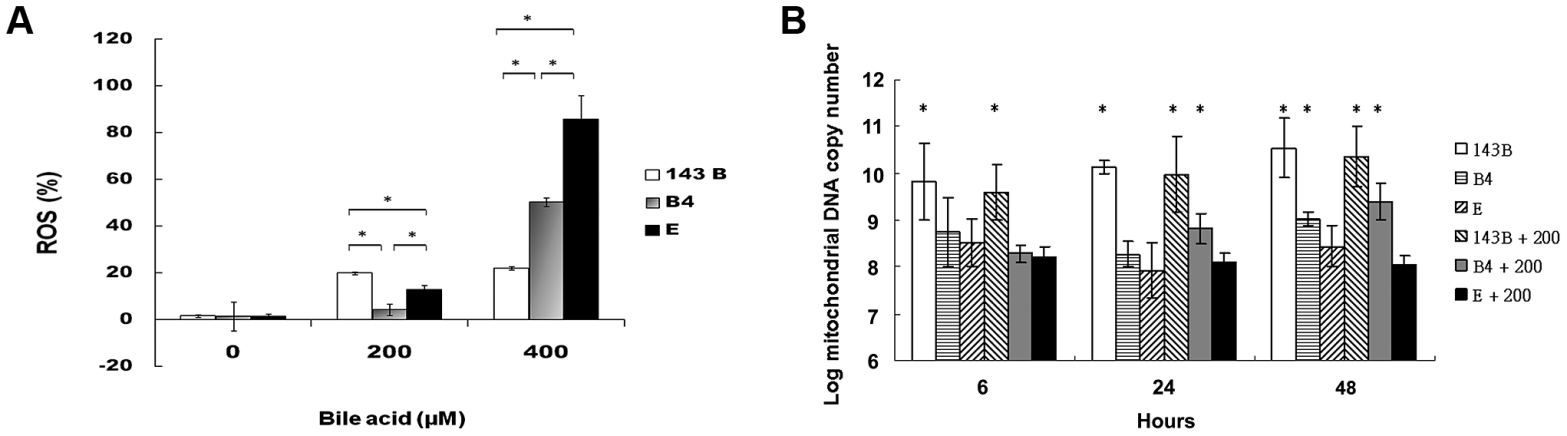
Measurement of reactive oxygen species (ROS) production and mitochondrial DNA copy number. (A) Flow cytometric measurement of reactive oxygen species in parental 143B cells, B4 and E cybrids after treatment with 0, 200 and 400 uM of bile acid for 24 h. * P<0.05 compared at the same doses of bile acid. (B) Changes of mitochondrial DNA copy number in parental 143B cells, B4 and E cybrids after treatment with 0 or 200 uM bile acid for 6, 24 and 48 h. The results represented were mean ± SE in six times tests * P<0.05 compared to cybrid E+200 uM of bile acid.

A flow cytometric measurement showed that the cybrid B4 cells had lower reactive oxygen species (ROS) production than the cybrid E and parental cells (Figure 2A).

mtDNA copy number usually measures the responsiveness of mitochondria to stress. In this respect, cybrid B4 was unique in that its mtDNA copy number increased significantly compared with those of the parental 143B and cybrid E cells at 24 and 48 h after the bile acid treatment. On the contrary, mtDNA copy number significantly decreased as early as 6 h after the bile acid treatment in the E cybrid cells compared with the parental 143B and B4 cybrid cells (Figure 2B).

### Less apoptosis in cells harboring haplogroup B4 than in parental or cybrid E cells treated with bile acid

**Figure 3:**
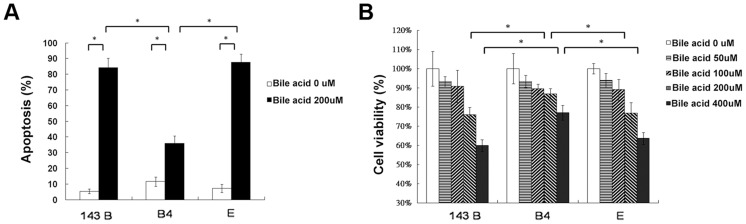
Cell apoptosis and viability. (A) Quantitation of flow cytometric measurement of apoptosis in parental 143B cells, B4 and E cybrids after treatment with 200 uM bile acid for 24 h and doubly stained with annexin V and PI. (B) The cell viability, measured by MTT assay, in parental 143B cells, B4 and E cybrids after treatment with 200 uM bile acid for 24 h. The results represented were mean ± SE in six times tests * P<0.05.

Hydrophobic bile acids such as chenodeoxycholic acid are generally toxic to the cells with bile acid transporters. To determine apoptosis, 143B cells and cybrid B4 and E cells were treated with different doses of chenodeoxycholic acid for 24 and 48 h. After treatment, annexin V and PI double staining was performed, revealing significantly less apoptosis in the cybrid B4 cells than in the cybrid E and parental cells (Figure 3A, after 24 h of treatment).

### Cell viability, as measured by MTT

(3-(4,5-Dimethylthiazol-2-yl)-2,5-diphenyltetrazolium bromide), indicated a dose-dependent decrease in viable cells among the 143Bcells, and cybrid B4 and E cells treated with increasing amount of bile acid. However, the cybrid E cells were most vulnerable to the bile acid treatment, having significantly fewer viable cells than the B4 and parental 143B cells at a dose higher than 200 µM for 24 and 48 h after the treatment (Figure 3B, after 24 h of treatment).

### Significantly higher mitochondrial fusion protein expression in cybrid B4 cells than in cybrid E and parental cells treated with bile acid

**Figure 4:**
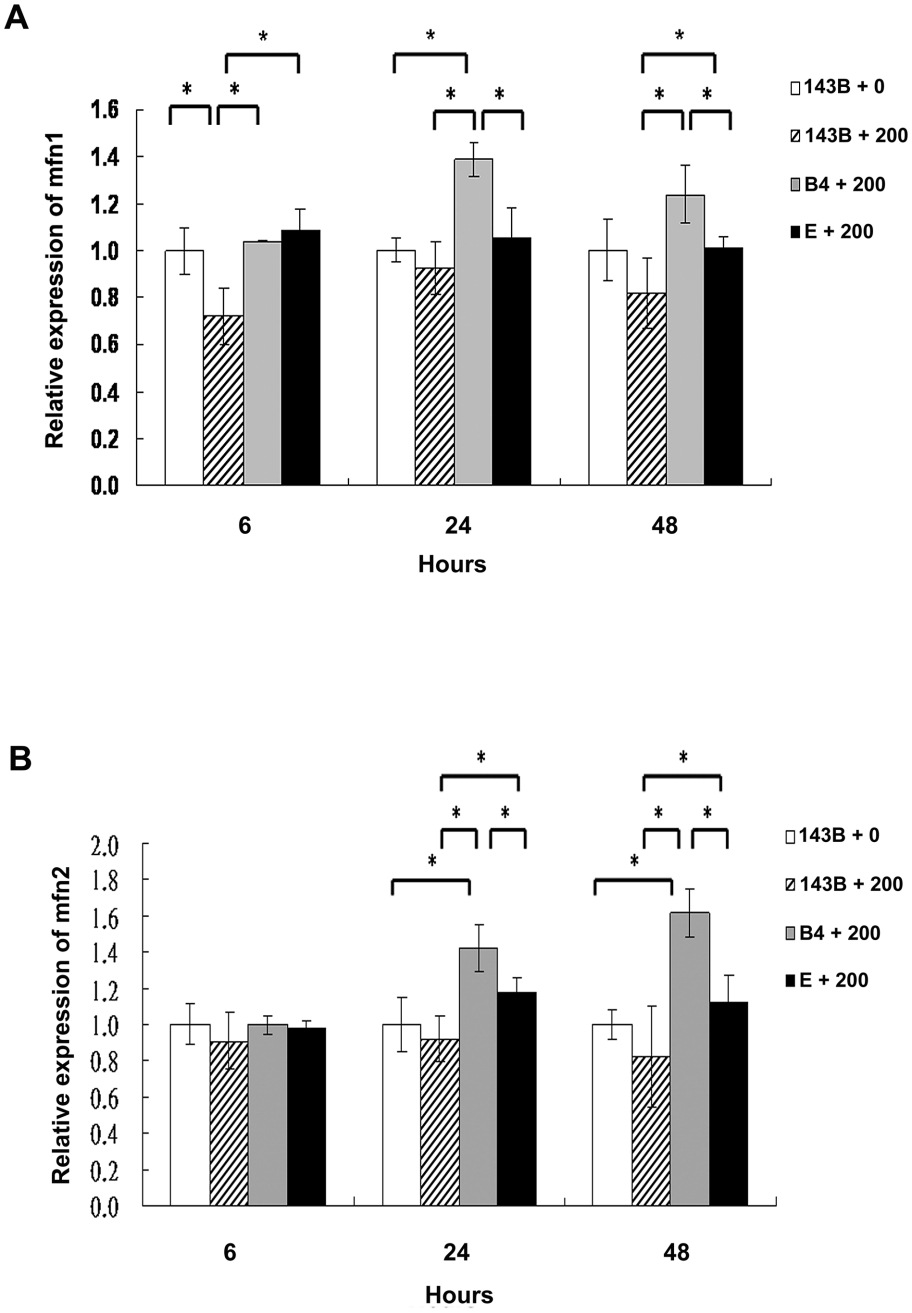
Analysis of the mitochondrial fusion molecules expression in the parental 143B cells, and cybrid B4 and E cells after the bile acid treatment. Western blot analysis of mitofusin 1 (mfn1) and mitofusin 2 (mfn2) expression in parental 143B cells, B4 and E cybrids after treatment with bile acid. (A) Histogram showed statistical difference between groups in mfn1 expression. Western blot of mfn 1 expression in parental 143B cells, B4 and E cybrids after treatment with 200 uM bile acid for 24 h and 48 h. (B) Histogram showed statistical difference between groups in mfn2 expression. Western blot of mfn 2 expression in parental 143B cells, B4 and E cybrids after treatment with 200 uM bile acid for 24 h and 48 h. The results represented were mean ± SE in six times tests * P<0.05.

Mitochondrial fusion is essential for organelle function and cellular homeostasis [19]. To assess mitochondrial fusion, the 143B cells, and cybrid B4 and E cells were treated with 200-µM chenodeoxycholic acid for 6, 24, and 48 h and were harvested to determine the mitofusin 1 (Mfn1) and mitofusin 2 (Mfn2) expression levels. As expected, significantly higher Mfn1 and Mfn2 expression levels were found in the cybrid B4 cells than in the cybrid E and parental 143B cells at 24 and 48 h after treatment (Figures 4A and 4B).

### Cybrid B4 exhibited better tolerance and respiratory function, and less glycolytic reaction following bile acid exposure than did cybrid E

**Figure 5:**
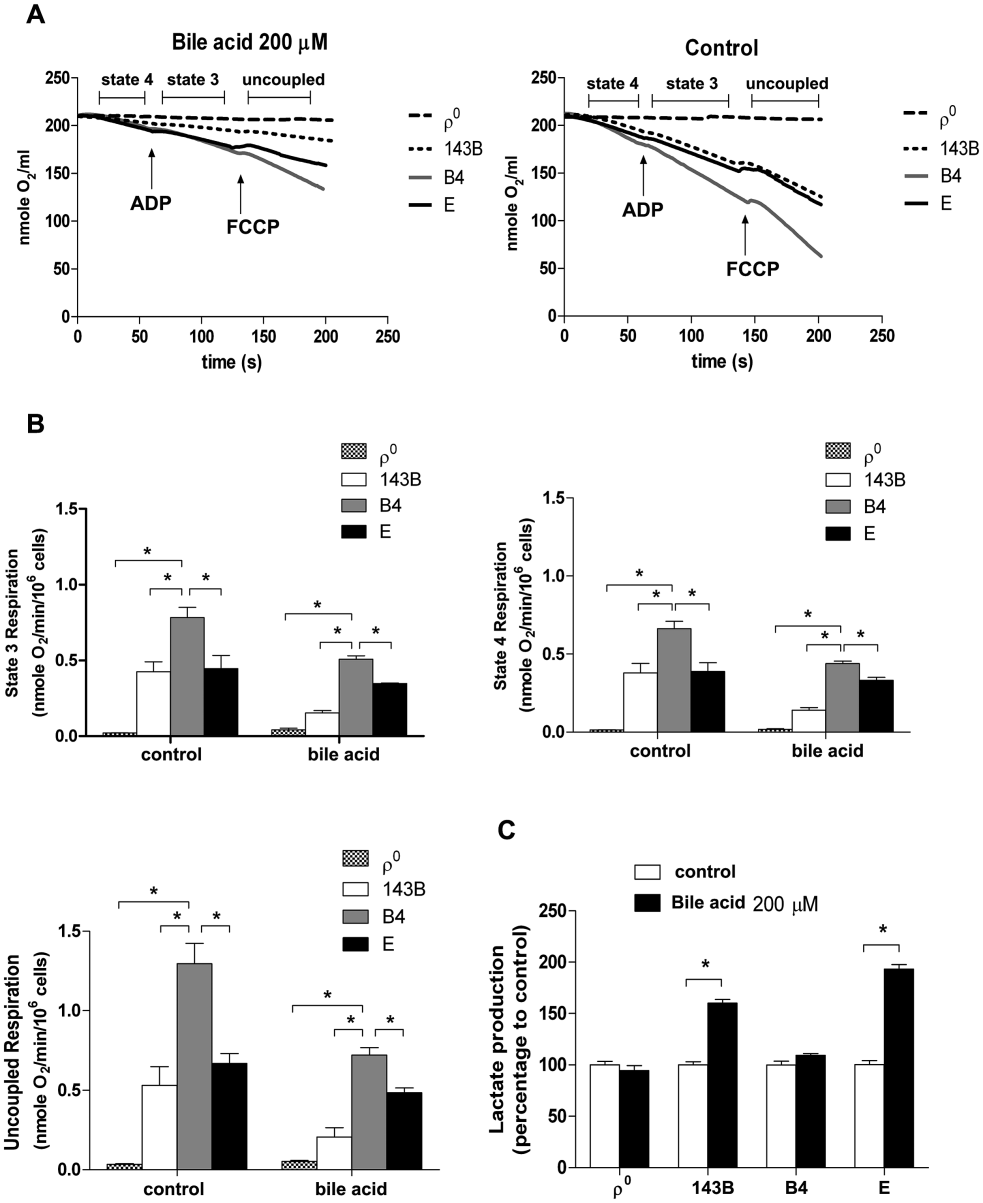
Effects of bile acid on OXPHOS and glycolysis in cybrid B4, cybrid E, parental 143B and ρ^0^ cell. Cybrid B4, cybrid E, parental 143B and ρ^0^ were treated with PBS or 200 µM bile acid (BA) for 24 h. (A) Measuring respiratory function, oxygen content in medium is illustrated by polarographic curves. Arrows indicated the point of adding drugs. Mitochondrial state 4 respiration was determined using 10 mM glutamate/malate as substrate. State 3 and uncoupled respiration were determined in the presence of 0.2 mM ADP and 1 µM FCCP respectively. 5×10^6^ cells/mL were resuspended in 100 µl respiratory medium. (B) Histogram showing the resulting oxygen consumption rate. (C) Culture medium was collected to detect the production of lactate, which was normalized to cell number. The results represented were mean ± SE in six times tests * P<0.05.

Our group has reported that cybrids with various mtDNA haplogroups demonstrated differences in oxidative phosphorylation (OXPHOS) capacity and viability under oxidative stress, suggesting mtDNA polymorphism contributes to differential tolerance against exogenous insult [20]. As shown in Figure 3B, cybrid B4 had better cell viability than cybrid E after exposure to bile acid. To assess whether the difference was due to different OXPHOS capacity and glycolysis, oxygen consumption rate (OCR) and lactate production were examined. Cybrid B4 had higher OCR in which ADP-limited (state 4), ADP-stimulated (state 3), and FCCP-induced (uncoupled) respiration than did cybrid E, 143B and ρ^0^ in either absence or presence of bile acid exposure (Figures 5A and 5B).In contrast, following bile acid exposure, cybrid E and parental 143B, but not cybrid B4 and ρ^0^, demonstrated a surge of lactate production (Figure 5C). These results indicated that the variations in tolerance against bile acid are correlated with mtDNA polymorphism-associated mitochondrial OXPHOS capacity. The differential viability and respiratory function in the presence of bile acid couple with metabolic shift to glycolysis.

## Discussion

In this study, we found that the mtDNA haplogroups B4 and E were associated with BA, although with entirely different implications. We found that whereas the haplogroup B4 was significantly less likely to occur in the patients with BA, the haplogroup E was more likely to occur in the patients with BA than in the healthy controls. Given that deregulation of mitochondrial function has been implicated in the pathogenesis of BA [1], [2], we studied the differential responses of cybrids B4 and E to a hydrophobic bile acid (chenodeoxycholic acid)-induced oxidative injury. As expected, we found that cybrid B4 maintained a significantly higher MMP and produced significantly less oxygen free radicals than the cybrid E and parental cells in response to the bile acid treatment. Furthermore, mitochondrial copy number and mitochondrial fusion protein expression, two protective responses of mitochondria to stress, were significantly higher in the cybrid B4 cells than in the cybrid E and parental cells, which was associated with significantly fewer apoptotic cybrid B4 cells than apoptotic cybrid E and parental cells. Overall, these findings suggested a protective effect of the haplogroup B4 from oxidative liver injury, which is at least in part due to inherently better mitochondrial function.

Consistent with our findings is a significantly higher evolutionary rate of mtDNA than that of nuclear DNA [21] because mtDNA is more vulnerable to high levels of ROS, lack of protective histones, and limited capacity for DNA repair than nuclear DNA [22]. Mitochondrial dysfunction has been linked to organ failures [23], [24], and polymorphisms in the mtDNA are expected to contribute more extensively to functional differences between individuals compared with polymorphisms in nuclear DNA [15]. It is not surprising that many human diseases have been associated with pathogenic mutations in the mtDNA or haplogroup-associated polymorphisms [16], [25], [26]. In particular, the sequence polymorphism in the D-loop region of the mtDNA was a risk factor for HCC [18]. The single nucleotide polymorphisms in the D-loop region of mtDNA 16266C/T, 16293A/G, 16299A/G, 16303G/A, 242C/T, 368A/G, and 462C/T minor alleles, were associated with increased risk for alcohol- HCC, and the 523A/del was associated with increased risks of alcohol- HCC and HBV-HCC [18].

Each mammalian cell contains several hundreds to more than a thousand mitochondria, and each organelle harbors 2–10 copies of mtDNA [27]. The mtDNA copy number in a specific cell type is usually precisely maintained but might be subjected to change during cell growth and differentiation [28]–[30]. In our study, the increased mtDNA copy number in the haplogroup B4 was a protective response, which is similar to the protective response of human beings against sepsis either through increased heat generation because of higher electron transport rates or looser coupling [31], or through ROS production to reduce infection [12]. About 96% of our population group, including the B4 cybrid line used in this study, carry T16217C but only 12% carry the additional A16247G, C16261T control region variants [32], [33]. The combinations of these variants are specifically identified as a Polynesian motif, which has also been reported in some of the Chinese population [32]. In our study (Table S1), there are eight mtDNA sequence alterations between B4 and E which cause amino acid changes. Four alterations occurred at subunits of respiratory complex I, three at complex IV and one change at complex V. Previously, mitochondrial complexes I and IV were suggested as being the main site of mitochondrial electron leakage [34]. Thus, it is feasible to hypothesize that amino acid alteration in the subunits of these enzyme complexes could lead to mitochondrial functional change, including a higher oxidative stress in haplogroup E, which is associated with significantly lower MMP, higher ROS production, and thus higher cellular apoptosis. The results also imply less likely occurrence of the haplogroup B4 in the patients with BA than in the haplogroup E.

Initial decreases in MMP and subsequent ROS production are a characteristic feature of pre-apoptosis [35]–[38]. In the present study, the bile acid treatment resulted in an initial significant decrease in MMP, which was associated with an excessive intracellular ROS production in the cybrid and parental cells in a time- and dose-dependent manner. The mtDNA-based differences, including different mtDNA and mtRNA levels, mitochondrial protein synthesis, cytochrome oxidase activity and level, normalized oxygen consumption, and mitochondrial inner membrane potential and growth capacity, were also found between cybrids [39]. Such inherited differences in mitochondrial function, including oxidative phosphorylation capacity [40], can help to explain the differential bioenergetic threshold toward oxidative liver injury between populations with different haplogroups that confer their different susceptibility to BA.

Occasional dysfunctional mitochondria can be complemented functionally through mitochondrial fusion. In cells lacking mitochondrial fusion, dysfunctional mitochondria may accumulate, leading to injury-induced cell death [39]. Mfn1 is the main mediator of mitochondrial fusion and homeostasis [41]. High Mfn1 expression levels significantly improved embryo development by increasing ATP levels and MMP while reducing H_2_O_2_ generation [41]. Mfn2 is also important in mtDNA transmission to daughter cells by tethering the mitochondrial membrane [42]. Downregulation of Mfn2 was found to induce cellular apoptosis, whereas overexpression of Mfn2 diminished cellular apoptosis [39]. In our study, we found that the cybrid B4 cells had higher Mfn1 and Mfn 2 expression levels than the cybrid E and parental cells in response to the bile acid treatment. The latter may also help to explain why apoptosis was significantly less in the cybrid B4 cells than in cybrid E and parental cells after the bile acid treatment.

Kim reported that synthetic bile acids induce apoptosis of osteosarcoma cells through a caspase and mitochondrial pathway [43]. Bile acid increases osteoblast differentiation, and neutralizes the detrimental effects of lithocholic acid in jaundiced patients [44]. In this study, the parental osteosarcoma cells harboring haplogroup X, after bile acid treatment, had different MMP, higher mtDNA copy number, different ROS production, higher cellular apoptosis and lower Mfn1 and Mfn 2 expression levels. We cannot exclude the differential effects of the hapogroup B4 and E from “wild type” as related to their mtDNA haplogroup, as well as to the change of the characteristics of osteosarcoma cells during the process of constructing cybrids.

From the findings that the patients with BA harboring haplogroup E had worse prognosis than other haplogroups and had to receive liver transplant earlier or else died, it is reasonable to check haplogroups in every BA case in the future to predict the outcomes as early as possible.

In conclusion, the study provides a novel insight into the etiopathogenesis of BA, based on the genetic background of mitochondria. The use of the cybrid models helped to establish the association of haplogroups B4 and E with BA and to clarify the population-based differences in the incidence of BA.

## Materials and Methods

### Patients and ethics statement

The study was approved by the Ethics and Clinical Research Committee of the Chang Gung Memorial Hospital (No. 97-2268A3). Seventy-one patients with BA and 200 healthy controls in the Taiwanese population of ethnic Chinese background were recruited from the Kaohsiung Chang Gung Memorial Hospital.

BA was confirmed on the basis of results of liver histological examination and intraoperative findings, which included 35 males and 36 females ranging in age from 0.1–11 years (median 2.7). All received screening for cytomegalovirus, rubella, herpes infection, but no specific causative factors were identified. As our hospital is the biggest referring center for liver transplantation in Taiwan, there are more patients referred for liver transplant after failed Kasai procedure in the other hospitals than receiving the operation primarily in this hospital. Accordingly, only eighteen patients survived with their native liver, while the remaining 53 patients either survived with liver transplant (46) or died (7).

The control group included 52 children and 148 adults. The 52 children were originally recruited for test of reaction to allergens and were proven negative for allergy. There were 25 males and 27 females, ranging in age from 0.3 to 16 years (medium 2.0). The adult population comprised 65 males and 83 females, ranging in age from 18 to 60 (medium 41). They were healthy Taiwanese subjects randomly selected from the health screening center. None of the subjects had a history of BA, cholestatic or autoimmune liver diseases (Table 1). The genetic background was similar in both control groups and was put together for statistical calculation. Informed written consent was obtained from their legal guardians or the participants for use of their blood in the study.

### Methods for determination of mitochondrial haplogroup

We performed haplogroup analysis, which included 40 mitochondrial single nucleotide polymorphisms in 15 major mitochondrial haplogroups. Genomic DNA was extracted from the whole blood. We used 24 pair primers to perform the gene amplification by multiplex polymerase chain reaction (PCR), which has been described as in Liou et al [32], [45]. The range of amplicon size was 190–300 base pairs (bp). We used 94 probes for mitochondrial haplogroup determination [32], [45]. The reactions were then measured by the Luminex100 flow cytometer. The detailed method for genotyping has been described in the protocol by Itoh Y et al [32], [45], [46].

### Genotyping of mtDNA

Venous blood (3 mL) was collected from each subject into tubes containing 50 mmol/L EDTA (disodium salt), and genomic DNA was isolated by use of a commercial kit (Genomix, Talent, Trieste, Italy) [32]. Mitochondrial polymorphisms were determined with sequence-specific oligonucleotide probes by the use of suspension array technology (Luminex 100; Luminex, Austin, TX). The details have been described as in Liou et al [32].

### Selection of mitochondrial polymorphism for haplogroup classification

The human mitochondrial single nucleotide polymorphism (mtSNP) database provided in the Mitomap website (http://www.mitomap.org/MITOMAP) was used for reference. We selected 40 mtSNPs that defined 15 major haplogroups (A, B, C, D, E, F, G, M7, M8, M9, M10, M11, M12, M13, N9) and their constitutive 13 sub-haplogroups (B4, B5, D4, D5, F1, F2, F3, F4, M7a, M7b, M7c, M8a, N9a) in this study. The selection was based on the previously constructed phylogenetic trees for Chinese and Japanese populations [32], [47], [48].

### Generation of ρ^0^ cells

Human osteosarcoma 143B cells were grown in Dulbecco's modified Eagle's medium (DMEM) containing 4.5 g/liter of glucose, supplemented with 10% (v/v) FBS, 50 units of penicillin/ml, and 50 µg/ml of streptomycin at 37°C in 5% CO2. For ρ^0^ cells generation, cells were grown in the presence of 50 ng/ml ethidium bromide in DMEM containing 4.5 g/liter of glucose, supplemented with 10% (v/v) FBS, 50 units of penicillin/ml, 50 µg/ml of streptomycin, 50 µg/ml of uridine, and 110 µg/ml of sodium pyruvate (ρ^0^ medium) at 37°C in 5% CO_2_ for three months. The medium was changed every 2–3 days and the cells were re-plated once a week. The absence of mitochondrial DNA was determined by real-time PCR or by Western blot confirmation of low or absent cytochrome c oxidase-II (Cox-II) expression [49].

### Construction of cybrids containing haplogroup B4 and E

Platelets were used as mtDNA donor. Fusion of platelets from the healthy Taiwanese volunteers with haplogroup B4 and E with 143B ρ^0^ cells were carried out in the presence of 50% (wt/vol) polyethylene glycol 1500 (Boehringer Mannheim). The fusion mixture was recovered for 1 week in ρ^0^ medium and then cultivated in selection DMEM medium, in which even cybrids with very low Cox-II expression were shown to grow without pyruvate and uridine. Negative control plates consisted of 143B ρ^0^ cells, which had undergone trans-mitochondrial fusion in the absence of platelets. On days 14–30 after fusion, successful cybrid clones growing in the medium were isolated clonally by limited dilution, while negative control cells died out. The presence of exogenously imported mtDNA, or specific mtDNA haplogroup in the cybrid was confirmed by Luminex100™ system.

### MTT assay

Human osteosarcoma 143B cells, 143B*ρ* ^0^ cells and cybrids carrying B4 and E haplogroups were washed once with phosphate-buffered saline (PBS), followed by the addition of 1 mL DMEM containing 0.05 mg/mL 3-(4,5-dimethylthiazol-2-yl)-2 and 5-diphenyltetrazolium bromide (MTT; Sigma). After incubation at 37°C for 1 h, the media were removed and the formazan crystals in the cells were dissolved in 1 mL DMSO. Cell viability was detected at an OD_570 nm_ using a spectrophotometer.

### Measurement of reactive oxygen species (ROS) production

To evaluate ROS levels, cybrid B4 and E cells (6×10^5^ cells/flask) were incubated with different concentrations of chenodeoxycholic acid for different time periods. Cells were then stained with 10 µM of Dichlorofluorescein diacetate for 30 min at 37°C, and then detached with trypsin/EDTA. Cells were collected in 1× PBS, washed twice by centrifugation (1500 rpm; 5 min), and resuspended in 0.5 mL 1× PBS for ROS production. It was measured by flow cytometry utilizing a BD Biosciences FACScan system. ROS production was expressed as mean fluorescence intensity (MFI), which was calculated by CellQuest software.

### Mitochondrial membrane potential (Δψ)

6×10^5^ cells were plated in six-well plates and allowed to attach for 16–18 h. After being treated with bile acids at different concentration for 24 h or 48 h, the cells were harvested by treatment with trypsin, washed in PBS, and resuspended in 200 ng/mL of Rhodamine 123 (Invitrogen). After incubation for 30 min at 37°C, the cells were washed three times and resuspended in 500 µL of PBS. Cytofluorimetric analysis was performed using a fluorescence-activated cell scanner machine (BD Biosciences FACScan system) [2], [49].

### Determination of mtDNA copy number

DNA samples were extracted from cybrid cells. The mtDNA copy numbers were measured by a real-time PCR and corrected by simultaneous measurement of the nuclear DNA (β-actin gene). The forward and reverse primers complementary to the nuclear β-actin gene and ND1 gene were as presented before [2]. The threshold cycle number (Ct) values of the β-actin gene and the ND1 gene were determined for each individual quantitative PCR run. Relative copy number (R_c_) = 2^ΔCT^, where ΔCT was the Ct _ß-actin_ - Ct _ND1_ [50]. Each measurement was carried out at least three times and normalized in each experiment against a serial dilution of a control DNA sample.

### Flow cytometric measurement of cell apoptosis

The cybrid B4 and, E cells (6×10^5^ cells/flask) were incubated with various concentrations of bile and H_2_O_2_ and harvested at 6, 24 and 48 h respectively. The percentage of cells in different phases of cell cycle was analyzed by flow cytometry after staining with propidium iodide (PI). Methodology is described briefly as follows; cells were fixed overnight with 70% ethanol at 4°C and washed twice with PBS. Then cells were centrifuged 1,500 RPM for 5 min and supernatant discarded. The cell pellet was resuspended in 1 ml PI (50 µg/ml)/0.05%Triton X-100 staining solution with RNase A (0.1 mg/ml) and incubated for 30 min at 37°C. Additionally, an apoptosis assay was performed using an Annexin V-FITC Apoptosis Detection Kit (BD Biosciences). The samples were analyzed on a Becton Dickinson FACScan flow cytometer using the CellQuest software. Annexin V positive/PI negative and Annexin V positive/PI positive cells were defined as necrotic and apoptotic cells respectively.

### Western blotting analysis

The cells were homogenized in a buffer then centrifuged at 14,000 g. Protein (40 µg) from the supernatant of each sample was separated by SDS-PAGE and transferred to polyvinylidene difluoride membranes for electrophoresis. The membranes were blocked in TBST buffer for 1 h at room temperature after which the blots were incubated with a primary cytochrome c oxidase-II (Cox-II), OATP (organic anion-transporting polypeptide), Mfn1, Mfn2 antibody (Santa Cruz Biotech.), followed by a secondary alkaline phosphatase-conjugated anti-IgG antibody (1∶5000; Promega). The Western blots were visualized with the Blot AP System (Promega). Alpha-tubulin or b-actin was used as internal control.

### Oxygen consumption analysis

Detection of oxygen consumption in permeabilized cells was described previously with some modifications [51], [52]. Oxygen consumption was monitored by a Clark electrode (Mitocell S200 micro respirometry system; Strathkelvin Instruments, Motherwell, UK). Cells (100 µL at 5×10^6^ cells/mL) in a respiratory medium (100 mM KCl, 3 mM MgCl_2_, 20 mM HEPES, 1 mM EDTA, and 5 mM KH_2_PO_4_; pH 7.4) were permeabilized by digitonin (optimal concentration 32.5 µg/mL as determined by trypan blue staining) and loaded into a 200 µL MT200 Respirometer chamber, suspended by a fixed-speed solid-state magnetic stirrer inside the chamber and maintained at 37°C by a circulating water bath. Mitochondrial state 4 respiration was determined using 10 mM glutamate/malate as substrates of respiratory complex I in the absence of ADP. Subsequently, mitochondrial state 3 was measured in the presence of 0.2 mM ADP. The following uncoupled respiration was determined by the addition of 1 µM carbonyl cyanide 4-(trifluoromethoxy)phenylhydrazone (FCCP) to the respiratory medium. Oxygen level in medium was expressed in nanomoles of O_2_ per mL. Oxygen consumption rate was expressed in nanomoles of O_2_ per minute per 10^6^ cells. Lactate was measured in collected culture medium using the Lactate Colorimetric Assay Kit (Abcam).

### Statistical analysis

Frequencies of all mitochondrial haplogroups in the BA patients and controls were tested for independency using Pearson chi-square statistics and Fisher's exact test as appropriate. Cybrid data were expressed as mean ± SE. Comparisons between different cybrids and parental cells were analyzed by the ANOVA with Bonferroni's correction when multiple comparisons were evaluated. We performed multivariate logistic-regression analysis, with BA as a dependent variable and independent variables including age and sex. Patient survival was assessed using the Kaplan–Meier method and compared between groups using the log–rank test. Survival with native liver was analyzed using time from birth until the time of follow-up excluding those who died or received liver transplantation. A p-value <0.05 was considered statistically significant. The statistical analyses were performed using the Statistical Package for Social Science (SPSS, version 12) software package.

## Supporting Information

Figure S1 Characteristics of 143B*ρ* ^0^ cells and B4 and E cybrids. (A) Absence of cytochrome c oxidase-II (Cox-II) protein, which is characteristic of depletion of mitochondrial DNA, was found in 143B*ρ* ^0^ cells but not in B4 and E cybrids. The latter also confirmed the successful introduction of mitochondria into B4 cybrid and E hybrid cells. (B) mRNA expression of organic anion-transporting polypeptide (OATP) in B4 and E cybrids was confirmed by using real-time quantitative RT-PCR. Effect of bile acid (chenodeoxycholic acid, 200 µM) on the expression OATP mRNA was also shown. The results represented were mean ± SE in six times tests. * indicates P<0.01 compared to control. (C) Western blot analysis revealed presence of OATP proteins in B4 and E cybrids and the increased levels of OATP in response to bile acid treatment. pc: Positive controls, obtained from the liver tissue of a patient with liver cirrhosis. * indicates P<0.01 compared to cybrid E+200 µM bile acid at the same time.
(TIF)

Table S1 Full-length sequences of the haplogroups B4 and E.
(DOC)
