# Supplementary figures and images for: Associations of Mitochondrial Haplogroups B4 and E with Biliary Atresia and Differential Susceptibility to Hydrophobic Bile Acid
Source: PLoS Genet. 2013 Aug 15;9(8):e1003696. doi: 10.1371/journal.pgen.1003696 (PMC3744426; doi:10.1371/journal.pgen.1003696)

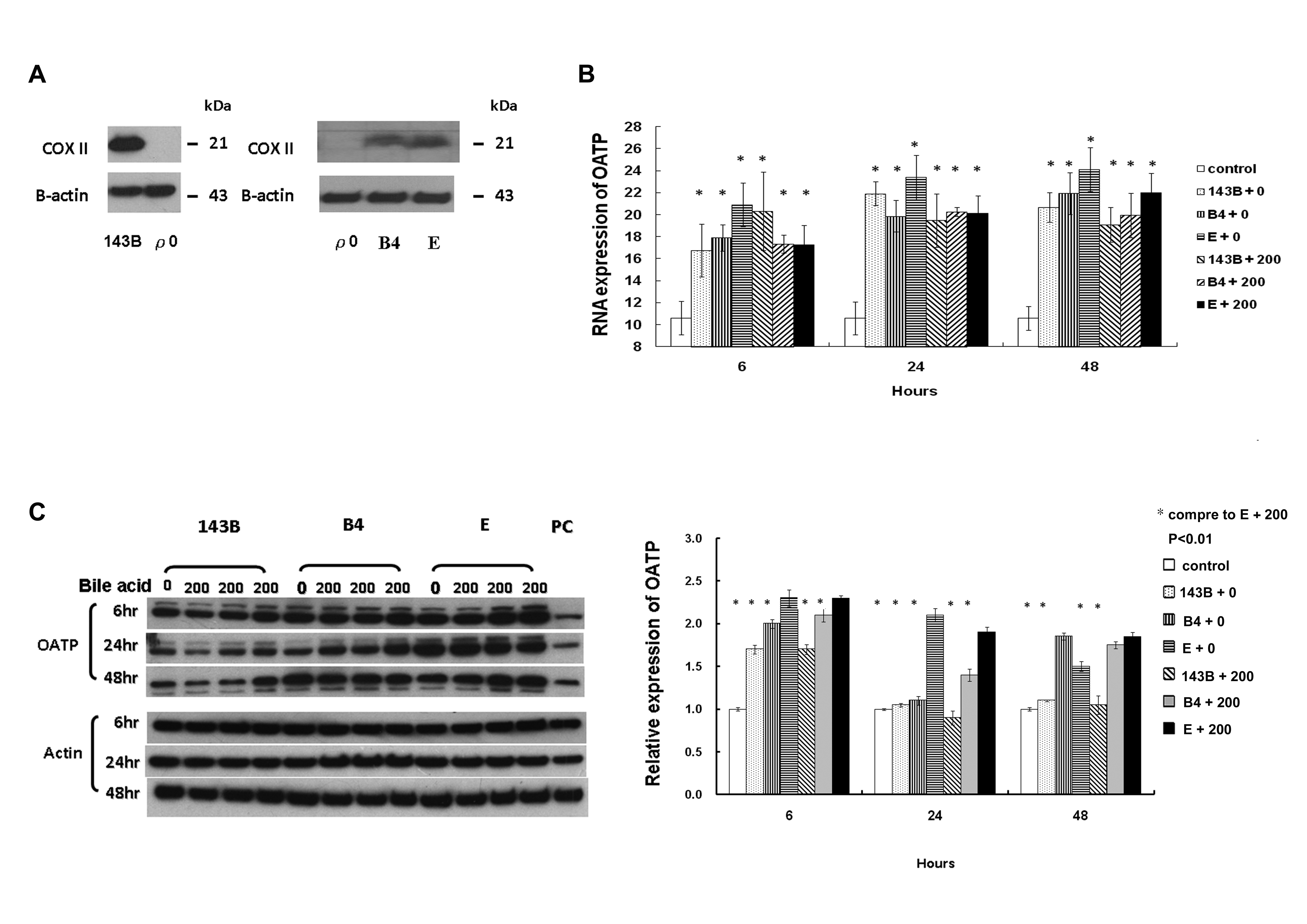

Supplement: Figure S1 — Characteristics of 143Bρ 0 cells and B4 and E cybrids. (A) Absence of cytochrome c oxidase-II (Cox-II) protein, which is characteristic of depletion of mitochondrial DNA, was found in 143Bρ 0 cells but not in B4 and E cybrids. The latter also confirmed the successful introduction of mitochondria into B4 cybrid and E hybrid cells. (B) mRNA expression of organic anion-transporting polypeptide (OATP) in B4 and E cybrids was confirmed by using real-time quantitative RT-PCR. Effect of bile acid (chenodeoxycholic acid, 200 µM) on the expression OATP mRNA was also shown. The results represented were mean ± SE in six times tests. * indicates P<0.01 compared to control. (C) Western blot analysis revealed presence of OATP proteins in B4 and E cybrids and the increased levels of OATP in response to bile acid treatment. pc: Positive controls, obtained from the liver tissue of a patient with liver cirrhosis. * indicates P<0.01 compared to cybrid E+200 µM bile acid at the same time. (TIF) [file pgen.1003696.s001.tif]
