# Supplementary material for: Associations of Mitochondrial Haplogroups B4 and E with Biliary Atresia and Differential Susceptibility to Hydrophobic Bile Acid
Source: PLoS Genet. 2013 Aug 15;9(8):e1003696. doi: 10.1371/journal.pgen.1003696 (PMC3744426; doi:10.1371/journal.pgen.1003696)
Supplement: Table S1 — Full-length sequences of the haplogroups B4 and E. (DOC) [file pgen.1003696.s002.doc]

| **Nucleotide position** | **Locus** | **B4a** | **B4b** | **B4c** | **B4d** | **E** | **Amino acid change** |
| --- | --- | --- | --- | --- | --- | --- | --- |
| **Non- synonymous** |  |  |  |  |  |  |  |
| 4491 | ND2 | G | G | G | G | A | V 8 I |
| 4596 | ND2 | G | G | G | G | G | V 43 I |
| 5178 | ND2 | C | C | C | C | C | L 235 M |
| 5301 | ND2 | A | A | A | A | A | I 277 V |
| 5466 | ND2 | A | A | A | A | A | T 333 A |
| 6253 | COI | T | T | T | T | T | M 117 T |
| 6267 | COI | G | G | G | G | G | A 122 T |
| 7266 | COI | G | G | G | T | T | S 455 A |
| 7598 | COII | G | G | G | G | A | A 5 T |
| 7934 | COII | A | A | A | A | G | I 117 V |
| 8414 | ATPase8 | C | C | C | C | C | L 17 I |
| 8584 | ATPase6 | G | G | G | G | G | A 20 T |
| 8684 | ATPase6 | C | C | C | C | C | T 53 I |
| 8701 | ATPase6 | A | A | A | A | G | T 59 A |
| 8860 | ATPase6 | G | G | G | G | G | T 112 A |
| 9053 | ATPase6 | G | G | A | G | G | S 176 N |
| 9099 | ATPase6 | C | C | C | C | C | I 191 M |
| 9128 | ATPase6 | T | T | T | T | T | I 201 T |
| 10084 | ND3 | T | C | T | T | T | I 9 T |
| 10398 | ND3 | A | A | A | A | G | T 114 A |
| 10400 | ND3 | C | C | C | C | T | T 114 A |
| 10609 | ND4L | T | T | T | T | T | M 47 T |
| 11969 | ND4 | G | G | G | G | G | A 404 T |
| 12026 | ND4 | A | A | A | A | A | I 423 V |
| 12338 | ND5 | T | T | T | T | T | M 1 T |
| 12358 | ND5 | A | A | A | A | A | T 8 A |
| 12406 | ND5 | G | G | G | G | G | V 24 I |
| 13135 | ND5 | G | G | G | G | G | A 267 T |
| 13708 | ND5 | G | G | G | A | G | A 458 T |
| 13759 | ND5 | G | G | G | G | G | A 475 T |
| 13834 | ND5 | A | A | A | A | A | T 500 A |
| 13928 | ND5 | G | G | G | G | G | S 531 T |
| 13942 | ND5 | A | A | A | G | A | T 536 A |
| 13966 | ND5 | A | A | A | A | A | T 544 A |
| 14318 | ND6 | T | T | T | T | T | N 119 S |
| 14577 | ND6 | T | T | T | T | C | I 33 V |
| 14751 | Cytb | T | C | C | C | C | T 2 I |
| 14766 | Cytb | C | C | C | C | C | I 7 T |
| 14979 | Cytb | T | T | T | T | T | I 78 T |
| 15204 | Cytb | T | T | T | T | T | I 153 T |
| 15326 | Cytb | G | G | G | G | G | T 194 A |
| 15758 | Cytb | A | G | A | A | A | I 338 V |
| **Synonymous** |  |  |  |  |  |  |  |
| 47 | D-loop | G | G | G | G | G |  |
| 52 | D-loop | T | T | T | T | T |  |
| 53 | D-loop | G | G | G | G | G |  |
| 54 | D-loop | G | G | G | G | C |  |
| 73 | D-loop | G | G | G | G | G |  |
| 143 | D-loop | G | G | G | G | G |  |
| 150 | D-loop | C | C | C | C | C |  |
| 151 | D-loop | C | C | C | T | C |  |
| 152 | D-loop | T | T | C | T | T |  |
| 153 | D-loop | A | A | A | A | A |  |
| 183 | D-loop | A | A | A | A | A |  |
| 185 | D-loop | G | G | G | G | G |  |
| 193 | D-loop | G | A | A | A | A |  |
| 195 | D-loop | T | T | T | T | T |  |
| 200 | D-loop | A | A | A | A | A |  |
| 210 | D-loop | A | A | A | A | A |  |
| 225 | D-loop | G | G | G | G | G |  |
| 226 | D-loop | T | T | T | T | T |  |
| 235 | D-loop | A | A | A | A | A |  |
| 248 | D-loop | A | A | A | A | A |  |
| 249 | D-loop | A | A | A | A | A |  |
| 263 | D-loop | G | G | G | G | G |  |
| 297 | D-loop | A | A | A | A | A |  |
| 309-315 | D-loop | CTCCCCCCC | CCTCCCCCC | CTCCCCCCC | CTCCCCCC | CTCCCCC |  |
| 310 | D-loop |  |  |  |  |  |  |
| 311 | D-loop |  |  |  |  |  |  |
| 312 | D-loop |  |  |  |  |  |  |
| 316 | D-loop |  |  |  |  |  |  |
| 317 | D-loop |  |  |  |  |  |  |
| 318 | D-loop | T | T | T | T | T |  |
| 414 | D-loop | T | T | T | T | T |  |
| 456 | D-loop | C | C | C | C | C |  |
| 489 | D-loop | T | T | T | T | C |  |
| 499 | D-loop | G | A | G | G | G |  |
| 514 | D-loop | - | - | - | C | C |  |
| 515 | D-loop | - | - | - | A | A |  |
| 522 | D-loop | C | C | C | C | C |  |
| 523 | D-loop | A | A | A | A | A |  |
| 556 | D-loop | A | A | A | T | A |  |
| 574 | D-loop | A | A | A | A | A |  |
| 678 | 12S rRNA | T | T | T | T | T |  |
| 681 | 12S rRNA | T | T | T | T | T |  |
| 709 | 12S rRNA | A | G | G | G | G |  |
| 749 | 12S rRNA | G | G | G | G | G |  |
| 752 | 12S rRNA | C | C | C | C | C |  |
| 827 | 12S rRNA | A | G | A | G | A |  |
| 930 | 12S rRNA | G | G | G | G | G |  |
| 1005 | 12S rRNA | T | T | T | T | T |  |
| 1009 | 12S rRNA | C | C | C | C | C |  |
| 1027 | 12S rRNA | A | A | A | A | A |  |
| 1048 | 12S rRNA | C | C | C | C | C |  |
| 1107 | 12S rRNA | T | T | T | T | T |  |
| 1119 | 12S rRNA | T | T | C | T | T |  |
| 1438 | 12S rRNA | G | G | G | G | G |  |
| 1520 | 12S rRNA | T | T | T | T | T |  |
| 1541 | 12S rRNA | T | T | T | T | T |  |
| 1676 | 16S rRNA | A | A | A | A | A |  |
| 1719 | 16S rRNA | G | G | G | G | G |  |
| 1824 | 16S rRNA | T | T | T | T | T |  |
| 2177 | 16S rRNA | T | T | T | T | T |  |
| 2227 | 16S rRNA | - | - | - | - | - |  |
| 2404 | 16S rRNA | A | A | A | A | A |  |
| 2706 | 16S rRNA | G | G | G | G | G |  |
| 2763 | 16S rRNA | T | T | T | T | T |  |
| 2835 | 16S rRNA | C | C | C | C | C |  |
| 2887 | 16S rRNA | T | T | T | T | C |  |
| 3010 | 16S rRNA | G | G | G | G | G |  |
| 3027 | 16S rRNA | T | T | T | T | C |  |
| 3106 | 16S rRNA | - | C | C | C | - |  |
| 3107 | 16S rRNA | C | - | - | - | C |  |
| 3111 | 16S rRNA | A | A | A | A | A |  |
| 3206 | 16S rRNA | C | C | C | C | C |  |
| 3528 | ND1 | C | C | C | C | C | syn |
| 3537 | ND1 | A | A | A | A | A | syn |
| 3552 | ND1 | T | T | T | T | T | syn |
| 3705 | ND1 | G | G | G | G | A | syn |
| 3970 | ND1 | C | C | C | C | C | syn |
| 4086 | ND1 | C | C | C | C | C | syn |
| 4248 | ND1 | T | T | T | T | C | syn |
| 4715 | ND2 | A | A | A | A | A | syn |
| 4769 | ND2 | G | G | G | G | G | syn |
| 4820 | ND2 | G | A | G | G | G | syn |
| 4883 | ND2 | C | C | C | C | C | syn |
| 4985 | ND2 | A | A | A | A | A | syn |
| 5063 | ND2 | T | C | T | T | T | syn |
| 5108 | ND2 | T | T | C | T | T | syn |
| 5153 | ND2 | A | A | A | A | A | syn |
| 5231 | ND2 | G | G | G | G | G | syn |
| 5252 | ND2 | G | G | G | G | G | syn |
| 5417 | ND2 | G | G | G | G | G | syn |
| 5465 | ND2 | C | T | T | T | T | syn |
| 5895-5899 | Non-coding | CCCCC | CCCCC | CCCCC | CCCCC | CCCCC |  |
| 6023 | COI | G | A | G | G | G | syn |
| 6026 | COI | G | G | G | G | G | syn |
| 6179 | COI | G | G | G | G | G | syn |
| 6221 | COI | T | T | T | T | T | syn |
| 6293 | COI | C | T | T | T | T | syn |
| 6371 | COI | C | C | C | C | C | syn |
| 6374 | COI | T | T | T | T | T | syn |
| 6392 | COI | T | T | T | T | T | syn |
| 6395 | COI | C | C | C | C | C | syn |
| 6413 | COI | T | C | T | T | T | syn |
| 6524 | COI | T | T | T | T | T | syn |
| 6620 | COI | T | T | T | T | C | syn |
| 6960 | COI | C | C | C | C | C | syn |
| 6962 | COI | G | G | G | G | G | syn |
| 7028 | COI | T | T | T | T | T | syn |
| 7196 | COI | C | C | C | C | C | syn |
| 7492 | tRNA Ser | C | C | C | C | C |  |
| 7828 | COII | A | A | A | A | A | syn |
| 7849 | COII | C | C | C | C | C | syn |
| 7912 | COII | G | G | G | G | G | syn |
| 8149 | COII | A | A | A | A | A | syn |
| 8271-8279 | Non-coding | --------- | --------- | ACCCCCTCT | --------- | ACCCCCTCT |  |
| 8281-8289 | Non-coding | ACCCCCTCT | ACCCCCTCT | --------- | ACCCCCTCT | ACCCCCTCT |  |
| 8473 | ATPase8 | T | T | T | T | T | syn |
| 8512 | ATPase8 | A | A | A | G | A | syn |
| 8718 | ATPase6 | A | A | A | A | A | syn |
| 8823 | ATPase6 | T | T | C | T | T | syn |
| 9123 | ATPase6 | A | G | G | G | G | syn |
| 9180 | ATPase6 | A | A | A | A | A | syn |
| 9536 | COIII | C | C | C | C | C | syn |
| 9540 | COIII | T | T | T | T | C | syn |
| 9545 | COIII | A | A | A | A | A | syn |
| 9548 | COIII | G | G | G | G | G | syn |
| 9950 | COIII | T | T | T | T | T | syn |
| 10310 | ND3 | G | G | G | G | G | syn |
| 10325 | ND3 | G | G | G | G | G | syn |
| 10397 | ND3 | A | A | A | A | A | syn |
| 10523 | ND4L | A | A | A | A | A | syn |
| 10535 | ND4L | T | T | T | T | T | syn |
| 10586 | ND4L | G | G | G | G | G | syn |
| 10834 | ND4 | C | C | C | C | T | syn |
| 10873 | ND4 | T | T | T | T | C | syn |
| 11050 | ND4 | T | C | T | T | T | syn |
| 11138 | ND4 | - | - | - | - | - |  |
| 11215 | ND4 | C | C | C | C | C | syn |
| 11254 | ND4 | T | C | T | T | T | syn |
| 11260 | ND4 | T | C | T | T | T | syn |
| 11335 | ND4 | C | C | C | C | C | syn |
| 11719 | ND4 | A | A | A | A | A | syn |
| 11914 | ND4 | G | G | G | A | G | syn |
| 11932 | ND4 | C | C | C | C | C | syn |
| 11944 | ND4 | T | T | T | T | T | syn |
| 12372 | ND5 | G | G | G | G | G | syn |
| 12666 | ND5 | A | A | A | A | A | syn |
| 12672 | ND5 | A | A | A | A | A | syn |
| 12705 | ND5 | C | C | C | C | T | syn |
| 12714 | ND5 | T | T | T | T | T | syn |
| 12717 | ND5 | C | C | C | C | C | syn |
| 12771 | ND5 | G | G | G | G | G | syn |
| 12882 | ND5 | C | C | C | C | C | syn |
| 12892 | ND5 | T | T | T | T | T | syn |
| 13044 | ND5 | C | C | C | C | C | syn |
| 13174 | ND5 | T | T | T | T | C | syn |
| 13194 | ND5 | G | A | G | G | G | syn |
| 13254 | ND5 | T | T | T | T | C | syn |
| 13263 | ND5 | A | A | A | A | A | syn |
| 13474 | ND5 | T | T | T | T | C | syn |
| 13590 | ND5 | G | A | G | G | G | syn |
| 13626 | ND5 | C | C | C | C | T | syn |
| 13695 | ND5 | C | C | C | C | C | syn |
| 13944 | ND5 | - | - | - | - | - |  |
| 14049 | ND5 | T | C | C | C | C | syn |
| 14088 | ND5 | T | T | C | T | T | syn |
| 14209 | ND6 | A | A | G | A | A | syn |
| 14241 | ND6 | A | A | A | G | A | syn |
| 14314 | ND6 | A | A | A | A | A | syn |
| 14470 | ND6 | T | T | T | T | T | syn |
| 14668 | ND6 | C | C | C | C | C | syn |
| 14783 | Cytb | T | T | T | T | C | syn |
| 14857 | Cytb | T | T | T | T | T | syn |
| 15034 | Cytb | A | A | A | A | A | syn |
| 15043 | Cytb | G | G | G | G | A | syn |
| 15172 | Cytb | G | G | G | G | G | syn |
| 15217 | Cytb | G | G | G | G | G | syn |
| 15235 | Cytb | A | A | A | A | A | syn |
| 15301 | Cytb | G | G | G | G | A | syn |
| 15310 | Cytb | T | T | T | T | T | syn |
| 15346 | Cytb | G | G | A | G | G | syn |
| 15397 | Cytb | A | A | A | A | A | syn |
| 15470 | Cytb | T | T | T | T | T | syn |
| 15481 | Cytb | C | C | C | C | C | syn |
| 15487 | Cytb | A | A | A | A | A | syn |
| 15535 | Cytb | C | T | C | T | C | syn |
| 15565 | Cytb | T | T | T | T | T | syn |
| 15688 | Cytb | C | T | C | C | C | syn |
| 15724 | Cytb | A | A | A | A | A | syn |
| 15894 | tRNA Thr | G | G | G | G | G |  |
| 15930 | tRNA Thr | G | G | G | A | G |  |
| 16092 | D-loop | T | T | T | T | T |  |
| 16093 | D-loop | T | T | T | T | T |  |
| 16108 | D-loop | C | C | C | C | C |  |
| 16126 | D-loop | T | T | T | T | T |  |
| 16129 | D-loop | G | G | G | A | A |  |
| 16136 | D-loop | T | C | T | T | T |  |
| 16140 | D-loop | T | T | T | T | T |  |
| 16147 | D-loop | C | C | T | C | C |  |
| 16148 | D-loop | C | C | C | C | C |  |
| 16162 | D-loop | A | A | A | A | A |  |
| 16164 | D-loop | A | A | A | A | A |  |
| 16167 | D-loop | C | C | C | T | C |  |
| 16172 | D-loop | T | T | T | T | T |  |
| 16182-16189 | D-loop | CCCCCCCC | ACCCCCCC | ACACCCCC | ACCCCCCC | AACCCCCT |  |
| 16217 | D-loop | C | C | C | C | T |  |
| 16223 | D-loop | C | C | C | C | T |  |
| 16234 | D-loop | C | C | C | T | C |  |
| 16235 | D-loop | A | A | G | A | A |  |
| 16249 | D-loop | T | T | T | T | T |  |
| 16257 | D-loop | C | C | C | C | C |  |
| 16260 | D-loop | C | T | C | C | C |  |
| 16261 | D-loop | T | C | C | C | C |  |
| 16266 | D-loop | C | C | C | C | C |  |
| 16274 | D-loop | G | G | G | G | G |  |
| 16278 | D-loop | C | C | C | C | C |  |
| 16288 | D-loop | T | T | T | T | T |  |
| 16293 | D-loop | A | A | A | A | A |  |
| 16298 | D-loop | T | T | T | T | T |  |
| 16299 | D-loop | G | A | A | A | A |  |
| 16304 | D-loop | T | T | T | T | T |  |
| 16309 | D-loop | A | A | A | A | A |  |
| 16311 | D-loop | T | T | T | T | T |  |
| 16319 | D-loop | G | G | G | G | G |  |
| 16325 | D-loop | T | C | T | T | T |  |
| 16327 | D-loop | C | C | C | C | C |  |
| 16357 | D-loop | T | T | T | T | T |  |
| 16362 | D-loop | T | T | T | T | C |  |
| 16390 | D-loop | G | G | G | G | A |  |
| 16519 | D-loop | C | C | C | C | C |  |
|  |  |  |  |  |  |  |  |
